# Chat-Based Decision Support System for the Maternal Health Journey in Assam, India: Protocol for a Mixed Methods Multiphase Implementation Study

**DOI:** 10.2196/81873

**Published:** 2026-02-13

**Authors:** Ashita Munjral, Swapnil Rawat, Charlotte E Warren, Jitender Nagpal, Ben Bellows, Aditi Aditi, Pompy Sridhar, Sowmya Ramesh

**Affiliations:** 1 Maternal Health, Population Council Consultancy, New Delhi, Delhi, India; 2 Research Department, Sitaram Bhartia Institute of Science and Research, B-16 Qutab Institutional Area, New Delhi, Delhi, 110016, India, 91 8860909873; 3 Social Behavioral Science Research, Population Council Inc, New York, NY, United States; 4 Department of Pediatrics, Sitaram Bhartia Institute of Science and Research, New Delhi, Delhi, India; 5 Digital Health, Nivi Inc., Sudbury, MA, United States; 6 Research Department, Population Council Institute, New Delhi, Delhi, India; 7 MSD For Mothers, MSD (India), Mumbai, Maharashtra, India

**Keywords:** maternal health, digital solution, protocol, Assam, India

## Abstract

**Background:**

Assam, India, has the country’s highest maternal mortality ratio (195 per 100,000 live births), mainly due to poor access to and quality of maternal health (MH) care. Many women receive inadequate antenatal and postnatal services, made worse by isolation, socioeconomic barriers, and weak health care infrastructure. Digital tools like mobile messaging and chatbots have improved antenatal care (ANC) and facility-based deliveries in similar settings. The e-SAATHI (Strengthening ANC/PNC via AskNivi Tailored Health Information, Referrals, and Follow-Up) project aims to provide personalized, stage-specific maternal health support through a chat-based system in Assam.

**Objective:**

This study assesses the acceptability, feasibility, and effectiveness of the e-SAATHI chatbot in increasing women’s access to MH information and improving ANC and PNC service uptake across public and private facilities. Objectives include increasing ANC/PNC use (eg, ≥4 ANC visits and timely PNC), promoting respectful care, and gathering insights for scaling digital health in high-burden regions.

**Methods:**

Phase 1 (0‐3 mo) involves co-designing and pilot testing aligned with World Health Organization and national guidelines. Phase 2 (4‐24 mo) involves enrolling pregnant and postpartum women via health facilities and social media. The chatbot sends 2-3 messages weekly from 10-week pregnancy to 15 weeks postpartum. About 300 health care providers will be trained and engaged for onboarding and feedback. Phase 3 (25‐36 mo) involves scaling up across districts, reaching 225,000 women. Data collection includes interviews, surveys, facility assessments, and chatbot analytics. Qualitative analysis will explore experiences; quantitative data (ANC completion, facility delivery, PNC follow-up, and satisfaction) will compare pre- and post-interventions. Ethical approvals, informed consent, and data confidentiality are observed.

**Results:**

The study was funded in September 2022. As of August 2025, 210 facilities have been onboarded, and 201,813 women were enrolled. Chatbot-based data collection began in April 2023 and will continue through the study period. Qualitative and quantitative evaluation data collection started in November 2023 and is expected to complete in June 2027. Interim analyses will be conducted after midline data collection in 2026; final analyses will be performed after endline data collection in 2027. The primary outcome will be the change in the composite quality score of maternal and newborn care. Secondary outcomes will include service uptake indicators, user-reported knowledge and self-care practices, and satisfaction with care. Operational feasibility—including provider integration and barriers such as digital literacy and connectivity—will also be assessed. Ongoing collaborative learning and adapting cycles are expected to capture intervention adaptations and inform optimal strategies for scale-up.

**Conclusions:**

e-SAATHI offers a scalable digital approach to improve MH across a variety of sociodemographic, linguistic, and risk settings. By delivering timely, personalized support, the chatbot may enhance health-seeking behavior and outcomes in Assam and in similar low-resource areas globally.

## Introduction

Despite decades of investments and initiatives to address intergenerational and deep-rooted social and cultural norms around maternal health (MH), progress has been slow in selected geographies of India, such as Assam. The state records the highest maternal mortality ratio in the country, nearly double the national average of 195 per 100,000 live births (2018‐2020) compared to 103 per 100,000 live births in (2017‐2019) [1-3]. Key indicators reflect significant gaps in care quality and utilization—only half of pregnant women receive the recommended 4 or more antenatal care (ANC) visits—approximately 48% received postnatal care (PNC) from trained health personnel, and, among Janani Suraksha Yojana beneficiaries, only 45% of newborns initiated breastfeeding within the critical first hour [4,5].

The persistently high maternal mortality rate in Assam stems from multiple interconnected factors [6,7]. Geographic barriers limit health care access, while socioeconomic challenges compound these difficulties [6,8]. The public health system faces an overwhelming delivery burden insufficiently coordinated with private MH providers [9]. Gaps include limited access to personalized pregnancy and childbirth information, inadequate self-care guidance, low insurance coverage, and high out-of-pocket expenditures [8,10,11]. Women’s restricted autonomy further constrains their access to, and use of, quality reproductive and MH services—collectively contributing to poor health outcomes for mothers and newborns [8,12]. While India has achieved a notable decline in maternal and newborn mortality over the past 2 to 3 decades, partly attributable to various maternal and newborn health policy reforms, significant challenges remain, particularly in high-burden states like Assam [6,7].

Direct-to-beneficiary mobile health (mHealth) programs that provide stage-based health information to new and expectant mothers have proliferated rapidly over the last decade [13]. In low-income and middle-income countries (LMICs), mHealth messaging programs represent some of the most successful examples of digital health programs, demonstrating measurable improvements in MH services [13,14]. Several digital MH services have achieved significant scale in Bangladesh, India, South Africa, and Tanzania, with each program reaching over 1 million subscribers and demonstrating potential for population-level impact [13,15].

The Government of India has prioritized digital health initiatives, leveraging the fact that more than 3-quarters of men and two-thirds of women own a mobile phone [16]. Smartphone ownership reaches approximately half of men (49%) and just over a quarter of women (26%) with notable variation between urban and rural settings.

Digital platforms and mHealth apps offer significant potential to improve the health information delivery and service access, even in contexts with low literacy levels [17-19]. Most digital health interventions use telephone-based systems that can engage individuals in their everyday lives, help them navigate into and across the health system, and capture client feedback following provider interactions.

Evidence demonstrates that messaging systems using digital apps or chatbots positively impact key MH indicators, including improved attendance at 4 or more ANC visits and increased facility-based deliveries [20-22].

This protocol paper describes the e-SAATHI (*Strengthening Antenatal Care/Postnatal Care via AskNivi Tailored Health Information, Referrals, and Follow-Up*) project—an iterative, collaborative learning and adapting (CLA) approach to document and monitor the introduction and scale-up of a chat-based digital support system (askNivi) for pregnant and postnatal women residing in the state of Assam, India, throughout their MH journey. Using WhatsApp, health information messages are sent to pregnant and postnatal women weekly based on the World Health Organization (WHO) guidelines during pregnancy and up to 15 weeks postnatally. The e-SAATHI project also identifies public and private MH providers to engage with the chatbot—to provide quality services to pregnant and postnatal women.

## Methods

### Aim and Objectives

The main aim of e-SAATHI is to evaluate the effectiveness of designing, implementing, and evaluating a digital health approach (chatbot) for improving women’s access to MH information and quality services in public and private health facilities in the state of Assam.

Key objectives are as follows: (1) to assess the acceptability and feasibility of an MH chatbot approach in increasing access/uptake of respectful MH services in public and private facilities; (2) to design, implement, monitor, and evaluate the effect of MH chatbot to improve the uptake of services; and (3) to document and assess the dynamics of implementing interventions to introduce an MH chatbot and generate lessons for replication/expansion at scale.

### Population

Our geographic target area is in Northeast India in the demographically and topographically diverse state of Assam where progress in improving key MH indicators has stalled and interventions are required to accelerate toward sustainable development goal targets. Given the challenges that Assamese pregnant women face regarding equitable access to timely, respectful, and quality MH care across geography and socioeconomic strata, a weak and overburdened public health system, and an underutilized private health sector, the e-SAATHI project intends to adapt, test, and scale a digital marketplace approach (chat-based digital support system: askNivi). Respectful maternity care is defined by WHO as care organized for and provided to all women in a manner that maintains their dignity, privacy, and confidentiality, ensures freedom from harm and mistreatment, and enables informed choice and continuous support during labor and childbirth [23]. This project will be implemented in up to 10 districts in the state of Assam, with a total of 300 health care facilities. Initially, 5 facilities will be brought on board, followed by additional facilities in 3 districts and finally facilities across 10 districts by the end of 3 years. For monitoring and evaluation purposes, care providers and users will both be participants for the qualitative work, while the chatbot users will be part of the quantitative dipstick surveys.

### Implementation

Understanding the urgent need to enhance MH outcomes in Assam, the Population Council Consulting Pvt. Ltd. (PCC), in collaboration with partners like Nivi Inc., Sitaram Bhartia Institute of Science and Research (SBISR), the Federation of Obstetric and Gynecological Societies of India, and the Centre for Northeast Studies and Policy Research, has launched the e-SAATHI initiative. This program aims to pilot and expand a chat-based digital support system for MH, designed to increase access to vital health information and strengthen maternal and newborn health across 10 districts in Assam through a comprehensive, multifaceted strategy.

e-SAATHI forms partnerships around a successful digital platform that empowers women to make informed choices about their reproductive health, promotes self-care, and connects users to reliable MH information and services throughout pregnancy and after childbirth. Given the obstacles pregnant women encounter in Assam, our approach is designed to both enhance and work alongside current public health programs related to MH. Most important, this solution helps reduce delays in seeking care by supporting women along their entire MH journey [24]. By maintaining engagement with expectant mothers from pregnancy through the postnatal period, the chat-based platform can help build greater awareness around healthy pregnancies (through timely and gestationally appropriate messages), encourage self-care practices, reinforce the value of ANC, facility-based births, and postnatal follow-up. It also facilitates timely connections with quality MH service providers, ensuring women get the safe and respectful care they need during this important period.

### Chatbot

**Figure 1.**
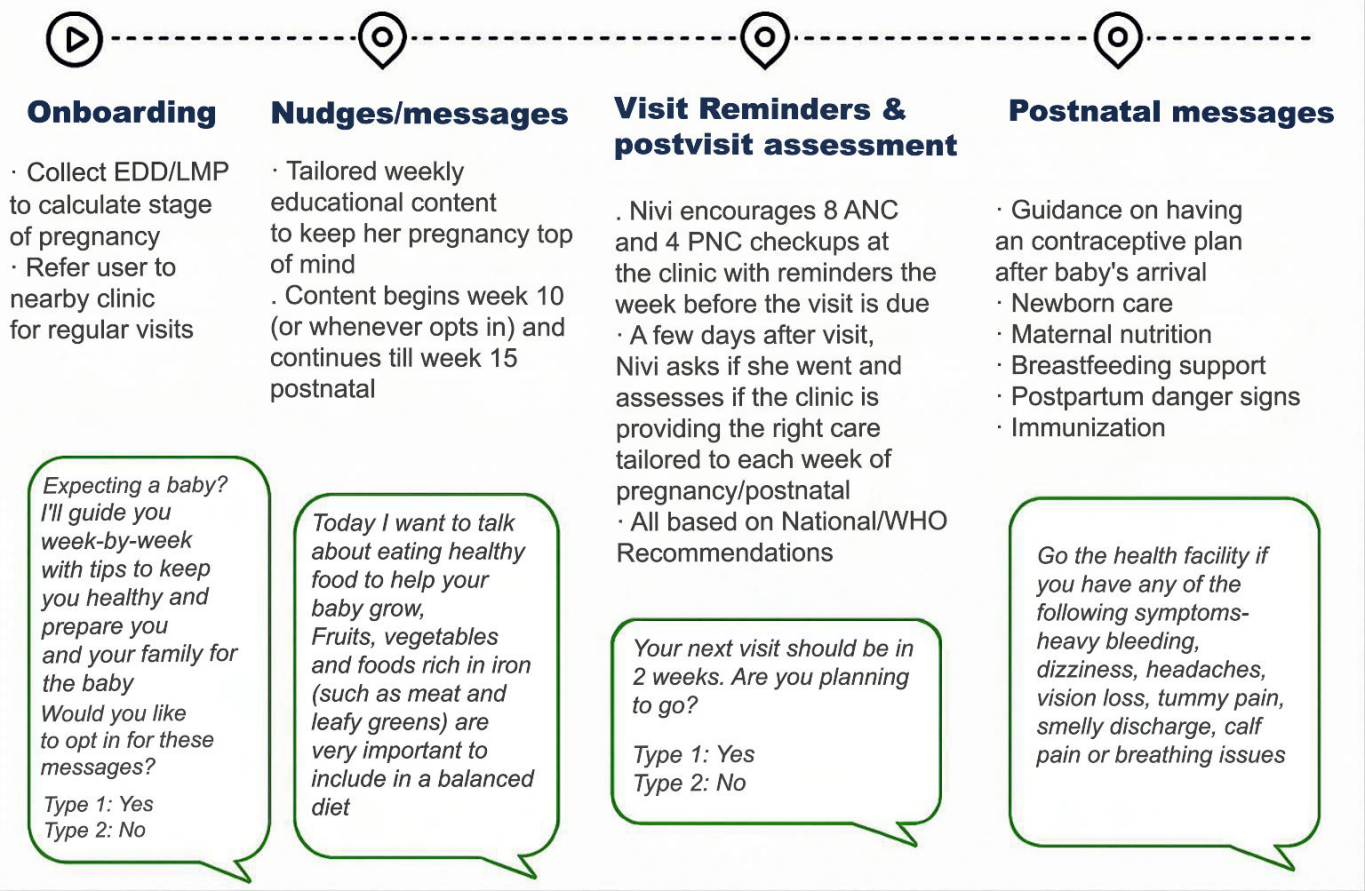
Proposed digital chat-based pathway for maternal health. ANC: antenatal care; EDD: expected date of delivery; LMP: last menstrual period; PNC: postnatal care; WHO: World Health Organization.

A key component of the e-SAATHI project is the chatbot messaging on MH information. It consists of a set of weekly tips and periodic reminders/check-ins that we send to subscribed users from prenatal week 10 through postnatal week 15. Women can subscribe to the free program in 1 of 2 ways: (1) clicking on a social media advertisement; or (2) scanning a QR code while visiting a health facility (public or private) that has engaged with the project. Both actions launch an automated conversation in WhatsApp that prompts the woman to onboard to the askNivi chatbot (accept terms and conditions and provide basic demographic information) and then subscribe to the ANC/PNC program (initially provide expected due date and preferred provider). Women who scan a QR code at a facility have the option of getting 1-on-1 support from a provider—typically a nurse—trained in user onboarding. The messages will be developed in alignment with the National Health Mission and WHO, ANC, and PNC guidelines (Figure 1).

The intervention will be implemented over 3 distinct phases: (1) establish and operationalize MH chatbot (0‐3 mo); (2) engagement, monitoring, and iterative improvement (4‐24 mo); and (3) scale-up (25‐36 mo).

### Phase 1: Establish and Operationalize MH Chatbot (January 2023-March 2023)

The MH chatbot will be developed in partnership with gynecologists, adhering to ANC and PNC guidelines established by the WHO and the National Health Mission. To ensure that all content aligns with current evidence and clinical standards, a diverse panel of health professionals will be convened. Additionally, a Women’s Ambassador Forum, comprising local community members—including pregnant and recently delivered women—will be formed to review the chatbot’s content for clarity, contextual relevance, and completeness. Upon completion of development, pilot testing will be conducted with a select group of approximately 15‐20 pregnant and postpartum women, including members of the Women’s Ambassador Forum, to assess message comprehension, cultural appropriateness, and usability. Feedback from users and subject matter experts will continually inform ongoing refinements to message wording, sequencing, and delivery as content is periodically updated.

We will collaborate with health care providers in both private and public hospitals to facilitate the enrollment of pregnant and postnatal women onto the digital system via the WhatsApp chatbot. Once enrolled, participants will receive 2 to 3 tailored messages per week, providing guidance aligned with their stage of gestation or weeks postnatal—including reminders for visits to designated MH providers—and offering opportunities to request additional support or submit feedback regarding their care. Additionally, this solution enhances MH provider workflows by using the MH digital support system. It streamlines onboarding processes, delivers automated reminders and follow-up prompts for both users and providers, and implements structured feedback mechanisms for ongoing improvement. These features collectively improve operational efficiency, enable prompt engagement, and ensure user feedback is systematically integrated into service delivery.

### Phase 2: Engagement, Monitoring, and Iterative Improvement (April 2023-September 2025)

#### Engagement

Onboarded women will be regularly engaged by health care providers or the research team whenever visiting the health facility to identify any challenges they face while using the chatbot. Additionally, feedback will be collected from users regarding the quality and usefulness of the services provided by the e-SAATHI chatbot.

#### Monitoring and Iterative Improvement

The monitoring and evaluation process will be conducted on users and health care providers. This study uses a mixed method, pre-post implementation design to evaluate the introduction and scale-up of the e-SAATHI MH chatbot in Assam. While this design enables comprehensive assessment of both quantitative outcomes and qualitative experiences, we acknowledge that the absence of randomization limits the strength of causal inference. To address potential sources of bias, we will implement several strategies, including adjustment for district-level clustering, accounting for secular trends over time, and controlling for measured confounders in the analysis. These approaches aim to strengthen the validity of our findings and provide a nuanced understanding of the intervention’s impact in real-world settings.

All the developed data collection instruments and procedures will be pretested in settings like those in which they will be administered. Guidance will be sought from postnatal women who have recently given birth when finalizing the instruments to ensure that questions and the data collection procedures are conducted with appropriate sensitivity and are not perceived as stigmatizing. The study tools will be pretested among a small group of women with characteristics similar to those of the study population to identify potential negative consequences and will be modified accordingly. Study tools for postnatal women will be translated and pretested in Assamese and Hindi.

The study comprises 5 main data collection tools that are described in Table 1: (1) in-depth qualitative interviews with MH providers; (2) in-depth qualitative interviews with postnatal women; (3) telephone interviews with postnatal women and MH chatbot users; (4) health facility inventory; and (5) MH digital platform program data.

**Table 1.** Data collection activities.^a^

	1. IDIs^b^ with health care providers	2. IDIs with postnatal (PN^c^) women	3. Survey with PN women	4. Facility inventory and routine data	5. MH^d^ data from chatbot platform
Study population	Purposively selected providers from various care levels (public and private)	Purposively sampled PN women using chatbot <6 mo since childbirth	PN women recently given birth in last 2‐8 wk	Facility providing MH services	Pregnant and PN women onboarded to chatbot (deidentified)
Sample size	10 from range of facility levels periodically	2‐4 women per facility—periodically	200 PN women	Each facility taking part in the project	All women who use chatbot
Location of activity	Private room at health facility or mutually agreed site	Private room at health facility or mutually agreed site	Recruitment at PNC^e^ ward then short interview (2‐8 wk later)	Facility level data (deidentified)	Online program data
Timing	Baseline and periodically 6‐9 mo	Baseline, midline, and endline	Baseline, then periodically (9 mo after chatbot introduction)	Quarterly	From initiation of chatbot to end of project
Method	In-depth face-to-face interview by researcher	In-depth face-to-face interview by researcher	Short in-person/telephone survey by researcher	Facilities visited by researcher	Analysis of chatbot program data

a Analysis of baseline, midline, and endline data will occur after the completion of each respective phase; interim analyses will be performed after midline (2026) and final analyses after endline (2027).

b IDI: in-depth interview.

c PN: postnatal.

d MH: maternal health.

e PNC: postnatal care.

First, this includes any MH provider (at least 10) over 18 years, who consent to be interviewed and work in one of the public or private facilities in the study areas, nurses, midwives, auxiliary nurse midwives, Accredited Social Health Activists (ASHAs), and doctors at health facilities engaged in the digital MH support system. The purpose of the interviews with MH providers is to identify facility and provider-specific issues that may affect the delivery of care and uptake of women onboarding onto the chatbot. Researchers will use a short in-depth interview (IDI) guide to ask respondents about their expectations regarding the MH chatbot, its strengths and weaknesses, develop an in-depth understanding of the process of introducing the chatbot, and how it might be improved and subsequently scaled up. Data will be analyzed iteratively after each collection wave to inform ongoing implementation and comprehensively the completion of phase 2.

Second, this will be conducted with a subsample of women (n=10) who used the MH chatbot and accessed its services in a facility. These interviews will be used to gain a deeper understanding of the motivations, perceptions, and priorities of women using the chatbot and facility services. Researchers will use a short IDI guide to ask respondents on their experience of using the MH chatbot, its strengths and weaknesses, and how it might be improved. Women will be at least 18 years old, have consented to the interview, and have given birth in a facility within the preceding 6 months. Data will be analyzed iteratively after each collection wave to inform ongoing implementation and the comprehensive completion of phase 2.

Third, postnatal women who have given birth and have experienced MH journey and/or used *askNivi* chatbot (>18 y age) will be approached in a postnatal ward at a facility. Women who consent to participate will be contacted telephonically between 2 to 8 weeks after the date of delivery for a telephonic or in-person survey. The study team will note their identifying information for future contact separately from the data. During the recruitment or enrollment at the facility, the research assistant will ask permission to contact the woman. A member of the study team will call and agree on a time that suits them. That same member of the team will then call to interview at the conveniently decided time. The short dipstick survey will cover the quality of care, experiences during pregnancy (number of ANC contacts, etc), childbirth and postnatally, and experience of using askNivi chatbot. Women who had loss or severe complications during childbirth were excluded. We will use the dipstick survey parameters field feedback to modify/adapt the composite quality score of maternal and newborn care as a composite measure designed to assess the quality of maternal and newborn health services received [24]. Each indicator will be scored based on whether the recommended standard will be met, typically using a binary (yes/no) scale. The scores for all indicators will then be summed to produce a total score, which will often be standardized to a percentage for ease of interpretation. This approach will allow for comparison of service quality across facilities and overtime and will help identify specific areas for improvement [24]. Quantitative analyses will be conducted after baseline, midline, and endline data collection points. Data cleaning and quality checks will occur fortnightly during collection with a comprehensive pass before midline and endline analyses.

Fourth, a short checklist including facility staffing levels; maternity unit capacity, medicines, supplies, and infrastructure; educational materials, job aids and protocols; health management information system and information and communication technology; facilitation and management of emergency referrals and transport; and other MH interventions available in the facility. This checklist will be administered to the in-charge or delegate of the facility. Statistics related to routine program data on utilization or uptake of maternal and newborn health services (ANC, PNC, births, and deaths) will be collected. Monthly trends in the numbers of new and continuing ANC and PNC clients will be obtained from facility records.

Fifth, this includes responses from women who have joined the MH chatbot (e-SAATHI), including demographic information and use of services. The data used is from all women who interact with the MH platform. Women who are on board with the chatbot permit their data to be used (deidentified).

The study uses a mixed methods approach to comprehensively document and monitor the rollout of the mental health chatbot for pregnant and postnatal women in Assam. Implementation is guided by continuous field feedback, data on onboarding and user engagement with the chatbot, the effectiveness of online campaigns, and qualitative and quantitative impact assessments. By adopting a participatory CLA methodology, the program integrates women’s perspectives and feedback from the earliest proof of concept, beginning in 5 facilities and then expanding across 3 districts to ultimately reach 10 districts within 3 years. These iterative CLA cycles, paired with regular evaluations of operational and financial sustainability, foster a deeper understanding of implementation challenges, including acceptability and feasibility. Upon completion of phase 2, a detailed impact evaluation will assess the effectiveness of the intervention, ensuring that crucial insights and real-world evidence are retained throughout the study’s progression. User engagement with the chatbot will be tracked as an automatically generated metric, supporting ongoing learning and adaptation to enhance the system. For the qualitative interviews and dipstick surveys, which are single, one-time interactions, with no follow-up assessments. However, we will systematically record and report the number of individuals who refuse participation or cannot be contacted at recruitment, along with reasons for refusal when volunteered. For the chatbot, we will also report the number of users who disengage before completing the interaction.

### Phase 3: Scale-Up and Sustainability (October 2025-June 2027)

In the final phase, the intervention will be scaled up to cover health care facilities across all 10 districts. This gradual expansion is planned over a period of 3 years, allowing for iterative refinement of the approach, capacity building of providers, and sustained engagement with stakeholders at each stage of implementation. The study population includes 300 MH providers and managers (nurses, midwives, auxiliary nurse midwives, doctors, and community health workers [ASHAs]) in selected private and public facilities across 10 districts in Assam with diverse geographic settings, as well as state-level managers or policymakers. We also anticipate reaching 225,000 pregnant/postnatal women through the chatbot.

### Data Management and Analysis

#### Overview

The data collection with postnatal women, providers, facility assessments, and service use statistics will be completed by trained research assistants using tablet computers. Upon completion of any electronic data collection form, all data will be immediately encrypted. Encrypted data cannot be decrypted without the unique private encryption key, to which only study investigators will have access. Encrypted survey data will be stored on research tablets until it can be transmitted to password-protected, study-specific servers using the local cellular data network. If cellular data service is unavailable at a given facility, the data collection team leader’s laptop computer will function as a central synchronization and backup point until the team reaches an area where mobile data service is available.

#### Qualitative Analysis

Qualitative data from the IDIs will be transcribed verbatim, translated into English where appropriate, and typed in Word. The data will be analyzed using Nvivo15 (Version 15; Lumivero) [25]. The analysis will entail open coding and progressive categorization of issues based on inductive (examining relevant issues that emerge from the data) and deductive approaches (examining key issues identified at the design stage). Themes will be further modified as more issues emerge from the data analysis. Categories derived from the data will be further refined through analysis charts. Hard copies of study documents will be stored under lock and key in secure drawers/rooms at PCC in New Delhi. We will upload digital recordings and store them on password-protected computers in folders on a secure network. We will store all data for at least 3 years before being destroyed by PCC.

#### Quantitative Analysis

Raw quantitative data collected from the postnatal women’s survey (dipstick survey) will be cleaned and checked for errors and outliers. Data cleaning and quality checks will occur fortnightly during collection with a comprehensive pass before midline and endline analyses. Descriptive statistics (means, SDs, medians, and frequencies) will summarize baseline characteristics and outcomes. The primary outcome, the composite quality score of maternal and newborn care [24] and secondary outcomes (service uptake indicators, user-reported knowledge and self-care practices, and satisfaction with care) will be compared between baseline and follow-up.

Normality will be tested (Shapiro-Wilk). Parametric (*t* tests, ANOVA) or nonparametric (Wilcoxon and Mann-Whitney) tests will be used as appropriate. Categorical variables will be analyzed using chi-square or Fisher exact test. Multivariable analyses will use generalized linear/logistic mixed models to adjust for clustering (facility/district) and confounders (age, parity, socioeconomic status, dose of exposure-engagement frequency, and enrollment channel). For repeated measures, mixed-effects models will assess changes over time. Multiple comparisons will be adjusted using Bonferroni or FDR. All analyses will be performed in IBM SPSS Statistics (Version 29.0; IBM Corp) and R Core Team. Results will be visualized with tables and graphs. Statistical significance is set at *P*<.05, with effect sizes and 95% CIs reported.

Interim analyses will be conducted after midline data (Phase 2) collection completion in 2026; final analyses will be performed after endline data collection in 2027.

#### Data Confidentiality

Any personal, organizational, or identifier information will not be attached to the data, nor will it be linked to the reports in a way that is traceable to an individual. Specifically, no names will be included in the transcripts, facility, and village names (that may characterize a community) will be described vaguely as a “rural” or “peri-urban site.” Pseudonyms or numbers will be assigned in naming stored data files to protect respondents from being identified. During dissemination of any type, all data will be presented in aggregate form so that names and other identifying information of a respondent will not be exposed—for example, an IDI with a postnatal woman will be classified in these ambiguous terms. Similarly, an IDI with a health provider will be described without explicitly naming a facility or area in which the data was collected. All data will be collected under the regulation of the data protection and management guidelines provided by the Government of India.

### Ethical Considerations

The research protocol has been reviewed by key stakeholders and ethical clearance has been granted by the Population Council’s Institutional Review Board (517), and the SBISR Ethics Committee (F.2/SBISR-EC/PR 01‐23). All researchers and research assistants will be trained on the conduct of ethical procedures and will be monitored during field work by the coalition led by PPC/SBISR. Informed consent will be obtained separately for each study participant and each component. All participants will be given detailed information about the study, including aims and methods of study; institutional affiliations of the research; anticipated benefits, risks, and discomfort it may cause (expected to be minimal); follow-up of the study; expected duration of an interview; a participant’s choice not to answer any questions; that they have the right to abstain from participating in the study or to withdraw from it at any time, without reprisal; measures taken to ensure confidentiality and anonymity of information provided; the conduct of interviews in places chosen by a participant that may maximize audio privacy; and contact details of the study coordinator for any questions or concerns. All data will be stored in password-protected computer files. Hard copies of questionnaires and anonymized transcriptions will be stored securely in a locked cabinet, in accordance with the PCC and SBISR policy. No compensation will be provided to participants.

## Results

The e-SAATHI study was formally funded in September 2022, marking the commencement of project activities. Since the initiation of the program, significant progress has been achieved in both the onboarding of health care facilities and participant recruitment. As of December 2025, a total of 210 health facilities across Assam have been successfully integrated into the e-SAATHI network. A total of 201,813 women have been enrolled to receive MH support through the chatbot platform.

Chatbot-based data collection was launched in April 2023 and is scheduled to continue through June 2027, ensuring comprehensive coverage of the intervention period. The collection of qualitative and quantitative evaluation data began in November 2023, providing a robust foundation for assessing both user experiences and measurable outcomes.

Interim analyses are planned following the completion of midline data collection in 2026, which will offer early insights into the effectiveness and operational feasibility of the intervention. Final analyses are scheduled to take place after endline data collection in 2027, at which point the full impact of the e-SAATHI program will be evaluated and published. The primary outcome of interest is the change in the composite quality score of maternal and newborn care, serving as a key indicator of the intervention’s effectiveness. Secondary outcomes include a range of service uptake indicators, user-reported knowledge and self-care practices, and satisfaction with care received. The study will also assess operational feasibility, including the integration of providers into the digital system and the identification of barriers such as digital literacy and connectivity challenges.

As of the writing of this paper (December 2025), we are completing phase 2 and initiating in-depth impact evaluation.

## Discussion

### e-SAATHI’s Implications

The introduction of the e-SAATHI intervention represents a significant advance in the realm of MH care, particularly within low-resource settings such as Assam, India. Assam has the highest maternal mortality ratio in India at 195 per 100,000 live births, nearly double the national average [1]. Given this context, there exists an urgent need for innovative solutions to address systemic gaps and improve MH outcomes. mHealth interventions, including chatbots and digital communication platforms, have demonstrated promise in improving MH care use in LMICs [26]. The implementation of e-SAATHI aligns with these global priorities, offering tailored health information, decision-making support, and continuous engagement to pregnant and postpartum women through digital channels. The observed changes in the composite quality score of maternal and newborn care [24] and domains will provide robust evidence of improvements in the quality and comprehensiveness of MH services. Secondary outcomes, including increased service uptake and improved knowledge/self-care, will further support the effectiveness and scalability of the intervention.

The e-SAATHI protocol leverages global evidence on the efficacy of mHealth tools in promoting maternal care use. Meta-analyses have consistently highlighted that digital health interventions such as chatbots and mobile messaging platforms significantly enhance key MH metrics, including ANC visits and facility-based delivery rates [26]. As a chatbot-driven initiative, e-SAATHI addresses longstanding gaps in MH education and accessibility, particularly in underserved regions. By providing stage-specific health information tailored to users’ gestation periods, the intervention reinforces global efforts aimed at improving maternal knowledge, autonomy, and continuity of care [27].

### Global Context and Precedent for Digital MH Interventions

In LMIC contexts, the adoption of digital health interventions has proven effective in mitigating barriers to health care engagement [4]. South Asia, in particular, has witnessed the large-scale implementation of mobile messaging programs for MH. For instance, India’s Kilkari program delivered weekly audio messages related to maternal and child health to over 10 million subscribers, improving pregnancy-related knowledge and self-care practices among participants [28]. While challenges such as subscriber retention and equitable reach were reported, the program underscored the feasibility of deploying gestation-tailored messaging as a complement to routine antenatal education. Similarly, Bangladesh’s “Aponjon” initiative provided stage-specific information to pregnant women via mobile devices, contributing to improved care-seeking behaviors, though coverage gaps persisted in hard-to-reach areas [29]. These findings emphasize the critical role of interactive, stage-specific messaging in augmenting MH services.

Sub-Saharan African countries provide additional examples of successful mHealth interventions. Chatbot and mobile messaging services have been widely implemented to support maternal and neonatal health, demonstrating high user demand and acceptability. African Researchers Magazine, 2023 [30] highlights the transformative impact of health chatbots in improving care-seeking behaviors among underserved populations. Such global precedents reinforce the rationale for implementing e-SAATHI, which is grounded in established evidence and adapted to local needs.

### Feasibility and Public Health Significance

The feasibility of e-SAATHI is supported by the widespread penetration of mobile phones and growing accessibility to internet services in LMICs. In India, over 70% of women own mobile phones, with an increasing proportion gaining access to smartphones and basic internet services [26]. High mobile penetration rates in Africa and Latin America have similarly facilitated the scalability of digital MH interventions. The public health significance of such initiatives is substantial, as they address critical gaps in health systems constrained by limited human resources. Women in low-resource settings often experience brief, infrequent clinic visits and overburdened health care providers, leading to missed opportunities for counseling and education. Chatbot programs such as e-SAATHI partially bridge this gap by delivering timely and personalized health messages, encouraging engagement with ANC services, immunizations, and institutional deliveries [30]. By supporting informed decision-making and early care-seeking behaviors, e-SAATHI contributes to achieving national and global targets for maternal and neonatal mortality reduction.

Qualitative evidence from prior interventions suggests that digital messaging services provide psychological support and empowerment to users. Women perceive these tools as continuous sources of information, which improves their confidence in navigating pregnancy-related challenges [26]. e-SAATHI’s interactive features, including quizzes and 2-way prompts, are designed to enhance user engagement and memory retention, particularly among individuals with limited literacy.

### Addressing Digital Inclusion and Equity

Despite the promise of digital health solutions, challenges related to equity and inclusion must be addressed. Vulnerable subgroups, such as women with low literacy levels or those residing in remote areas without smartphones, may face barriers in accessing e-SAATHI’s services. These concerns are explicitly addressed in the intervention’s design. The chatbot platform is delivered via widely available messaging apps such as WhatsApp, ensuring accessibility for diverse user groups. Content is provided in local languages, including Assamese and Hindi, to maximize comprehension and cultural relevance. Collaborations with ASHAs and nurses further enhance trust and engagement among users. This blended approach mirrors the successful integration of digital and human-mediated services observed in South Africa’s MomConnect initiative [31,32].

Infrastructure obstacles, such as poor connectivity in rural areas, are mitigated through asynchronous messaging capabilities, allowing users to access content even in regions with intermittent internet access. By prioritizing inclusive design features, e-SAATHI ensures its accessibility to underserved populations, thereby enhancing its scalability and impact.

### Study Strengths and Limitations

#### Strengths

The e-SAATHI implementation stands out for its innovative use of a scalable, chat-based digital platform to deliver personalized MH information, a particularly valuable approach in resource-constrained regions like Assam where it addresses persistent gaps in MH education and access. The intervention was co-designed in partnership with local community members, health care providers, and national experts, ensuring that it is contextually relevant, culturally appropriate, and responsive to user needs. Using a mixed methods evaluation, the study integrates qualitative and quantitative methodologies to capture both measurable outcomes and nuanced experiences of users and providers, thereby enhancing the overall assessment of feasibility, acceptability, and effectiveness. Throughout the study, robust ethical and data security protocols—including thorough informed consent processes, stringent data confidentiality procedures, and comprehensive ethical oversight—safeguard participant privacy and uphold the integrity of all collected data.

#### Limitations

The implementation of the e-SAATHI intervention faces several limitations. First, digital access barriers remain a concern, as users in areas with limited or no internet connectivity may be unable to access the chatbot. Some pregnant and postnatal women may not have smartphones or internet access, potentially excluding those who most need information. Although mobile phone density is generally adequate in the study districts, alternative delivery mechanisms—such as dissemination through ASHAs or other media—are being explored to address this gap. Additionally, systemic and operational challenges could arise, as the alignment of partner facilities and district health officials with the intervention’s goals may vary, potentially causing delays. Microplanning differences in implementation could also result in varied onboarding and user experiences across sites. The study design presents another constraint; while the pre-post monitoring and evaluation framework is practical for real-world application, it limits the strength of causal inferences. Without randomization, observed changes may be influenced by external factors or secular trends, despite efforts to adjust for confounders and clustering. There is also the potential for selection bias, as women who are able to access and use the chatbot may differ systematically from those who cannot, which could affect the generalizability of the findings.

### Conclusion and Future Implications

The e-SAATHI intervention exemplifies a scalable and inclusive approach to improve MH outcomes in Assam and beyond. Its design reflects lessons from global experiences, emphasizing digital inclusion, cultural adaptation, and user engagement. While its effectiveness remains to be evaluated, the intervention demonstrates potential to complement existing MH initiatives, such as cash incentive programs and community health worker outreach. The implementation protocol includes mixed methods evaluations to capture quantitative metrics, user experiences, and integration within health systems. These insights will inform the scalability and sustainability of chatbot-based interventions for MH care.

By addressing persistent gaps in MH services, e-SAATHI contributes to the broader objective of equitable health care access. Its participatory design and evidence-based approach position it as a promising addition to the MH toolkit in India and similar LMIC contexts. Continued investigation and adaptation of the intervention will enhance its relevance and impact, advancing global efforts to improve maternal and neonatal health outcomes.

## Abbreviations

ANC: antenatal care
ASHAs: Accredited Social Health Activists
CLA: collaborative learning and adapting
e-SAATHI: Strengthening ANC/PNC via AskNivi Tailored Health Information, Referrals, and Follow-Up
IDI: in-depth interview
LMICs: low-income and middle-income countries
MH: maternal health
mHealth: mobile health
PCC: Population Council Consulting Pvt. Ltd.
PNC: postnatal care
SBISR: Sitaram Bhartia Institute of Science and Research
WHO: World Health Organization

## References

[1] Meh C, Sharma A, Ram U (2022). Trends in maternal mortality in India over two decades in nationally representative surveys. BJOG.

[2] Faridi SB, Selvam I (2022). Maternal health situation in Uttar Pradesh, India. Eras J Med Res.

[3] Purohit T, Purohit P, Satender C, Batwal P (2024). A correlation analysis between maternal health service indicators aggregate scoring and maternal mortality ratio: a quantitative study of states in India. SN Compr Clin Med.

[4] Kayaroganam R, Saya GK, Kar SS (2016). Utilization of maternal health services among Janani Suraksha Yojana beneficiaries in Puducherry, India. Int J Adv Med Health Res.

[5] Sen S, Chatterjee S, Khan PK, Mohanty SK (2020). Unintended effects of Janani Suraksha Yojana on maternal care in India. SSM Popul Health.

[6] Tripathi P, Chakrabarty M, Singh A, Let S (2024). Geographic disparities and determinants of full utilization of the continuum of maternal and newborn healthcare services in rural India. BMC Public Health.

[7] Bhushan H, Ram U, Scott K (2024). Making the health system work for over 25 million births annually: drivers of the notable decline in maternal and newborn mortality in India. BMJ Glob Health.

[8] Hamal M, Dieleman M, De Brouwere V, de Cock Buning T (2020). Social determinants of maternal health: a scoping review of factors influencing maternal mortality and maternal health service use in India. Public Health Rev.

[9] Purbey A, Kumar A, Mozumdar A, Mishra P, Acharya R, Saggurti N (2025). Trends in the utilisation of maternal and child healthcare services from the public and private health sectors in India, 2005-2021: an analysis of cross-sectional survey data. BMJ Open.

[10] Gebremedhin TA, Mohanty I, Niyonsenga T (2022). Public health insurance and maternal health care utilization in India: evidence from the 2005-2012 mothers’ cohort data. BMC Pregnancy Childbirth.

[11] Lusome R, Sivan A, Kumar MA (2025). Out of pocket expenditure on institutional deliveries in India. Matern Child Health J.

[12] Mondal D, Karmakar S, Banerjee A (2020). Women’s autonomy and utilization of maternal healthcare in India: evidence from a recent national survey. PLoS ONE.

[13] Scott K, Ummer O, Shinde A (2021). Another voice in the crowd: the challenge of changing family planning and child feeding practices through mHealth messaging in rural central India. BMJ Glob Health.

[14] Murthy N, Chandrasekharan S, Prakash MP (2019). The impact of an mHealth voice message service (mMitra) on infant care knowledge, and practices among low-income women in India: findings from a pseudo-randomized controlled trial. Matern Child Health J.

[15] Kabongo EM, Mukumbang FC, Delobelle P, Nicol E (2019). Understanding the influence of the MomConnect programme on antenatal and postnatal care service utilisation in two South African provinces: a realist evaluation protocol. BMJ Open.

[16] Ranganathan R (2020). Towards a holistic digital health ecosystem in india. https://www.orfonline.org/public/uploads/posts/pdf/20230524152812.pdf.

[17] Muthappan S, Elumalai R, Shanmugasundaram N (2022). AYUSH digital initiatives: harnessing the power of digital technology for India’s traditional medical systems. J Ayurveda Integr Med.

[18] Patel K, Nayak B, Behera TR (2022). Factors influencing, SWOT analysis and emerged strategies for improving the use of digital data platforms in immunisation program by frontline health care providers: a study from rural eastern India. Int J Community Med Public Health.

[19] Rasekaba TM, Pereira P, Rani G V, Johnson R, McKechnie R, Blackberry I (2022). Exploring telehealth readiness in a resource limited setting: digital and health literacy among older people in rural India (DAHLIA). Geriatrics (Basel).

[20] Bahja M, Abuhwaila N, Bahja J (2020). An antenatal care awareness prototype chatbot application using a user-centric design approach.

[21] Grenier L, Suhowatsky S, Kabue MM (2019). Impact of group antenatal care (G-ANC) versus individual antenatal care (ANC) on quality of care, ANC attendance and facility-based delivery: a pragmatic cluster-randomized controlled trial in Kenya and Nigeria. PLoS ONE.

[22] Rahman MO, Yamaji N, Nagamatsu Y, Ota E (2022). Effects of mHealth interventions on improving antenatal care visits and skilled delivery care in low- and middle-income countries: systematic review and meta-analysis. J Med Internet Res.

[23] (2018). WHO recommendations on intrapartum care for a positive childbirth experience. https://iris.who.int/server/api/core/bitstreams/ba043cf7-cba4-484d-bf7e-ec79c4102d54/content.

[24] Lee HY, Rana MJ, Kim R, Subramanian SV (2022). Small area variation in the quality of maternal and newborn care in India. JAMA Netw Open.

[25] (2025). NVivo (version 15). Lumivero.

[26] Ramesh S, Warren CE, Bellows B (2025). Leveraging health financing, digital health and self-care approaches to strengthen maternal health journeys in India: perspectives from Assam. Front Glob Womens Health.

[27] Muthelo L, Mbombi MO, Bopape MA (2023). Reflections on digital maternal and child health support for mothers and community health workers in rural areas of Limpopo Province, South Africa. Int J Environ Res Public Health.

[28] Mohan D, Bashingwa JJH, Scott K (2022). Optimising the reach of mobile health messaging programmes: an analysis of system generated data for the Kilkari programme across 13 states in India. BMJ Glob Health.

[29] Alam M, Banwell C, Olsen A, Lokuge K (2019). Patients’ and doctors’ perceptions of a mobile phone-based consultation service for maternal, neonatal, and infant health care in Bangladesh: a mixed-methods study. JMIR mHealth uHealth.

[30] Phiri M, Munoriyarwa A (2023). Health chatbots in Africa: scoping review. J Med Internet Res.

[31] Ochieng H, Awosiku O An overview of healthcare chatbots in Africa. Digital Health Africa.

[32] Verma T (2022). Using AI to improve maternal health chatbots in South Africa. IDinsight.

